# Understanding Traumatic Brain Injuries in Military Personnel: Investigating the Dynamic Interplay of the Cerebrospinal Fluid and Brain During Blasts

**DOI:** 10.7759/cureus.46962

**Published:** 2023-10-13

**Authors:** Elisabeth Frankini, Eric J Basile, Faiz Syed, Ong Chi Wei, Milan Toma

**Affiliations:** 1 Department of Osteopathic Manipulative Medicine, New York Institute of Technology College of Osteopathic Medicine, Old Westbury, USA; 2 Department of Internal Medicine, University of Florida, Gainesville, USA; 3 School of Chemistry, Chemical Engineering and Biotechnology, Nanyang Technological University, Singapore, SGP

**Keywords:** blasts, military, brain, cerebrospinal fluid, computational, fluid-structure interaction

## Abstract

Background

It is estimated that around 450,000 traumatic brain injury cases have occurred in the 21st century with possible under-reporting. Computational simulations are increasingly used to study the pathophysiology of traumatic brain injuries among US military personnel. This approach allows for investigation without ethical concerns surrounding live subject testing.

Methodology

The pertinent data on head acceleration is applied to a detailed 3D model of a patient-specific head, which encompasses all significant components of the brain and its surrounding fluid. The use of finite element analysis and smoothed-particle hydrodynamics serves to replicate the interaction between these elements during discharge through simulation of their fluid-structure dynamics.

Results

The stress levels of the brain are assessed at varying time intervals subsequent to the explosion. The regions where there is an intersection between the skull and brain are observed, along with the predominant orientations in which displacement of the brain occurs resulting in a brain injury.

Conclusions

It has been determined that the cerebrospinal fluid is inadequate in preventing brain damage caused by multiple abrupt directional shifts of the head. Accordingly, additional research must be undertaken to enhance our comprehension of the injury mechanisms linked with consecutive changes in acceleration impacting the head.

## Introduction

Acquired brain injury caused by external factors or forces resulting in enduring cognitive and behavioral symptoms characterizes traumatic brain injury (TBI). Incidents of TBI among military personnel may arise during or after their service. The documented cases of TBI across all branches of the US military from 2000 to Q3 2021 amount to almost 450,000, covering a spectrum ranging from mild to penetrating injuries [1]; however, given that many occurrences remain unreported, the actual figures could be higher [2]. According to estimates, the incidence of blast-related TBI among military personnel deployed in Iraq and Afghanistan during Operations Iraqi Freedom and Enduring Freedom is high, ranging between 19% and 23% [3]. While patients with mild TBI typically recover within a few months, others may experience persistent symptoms known as post-concussive syndrome (PCS), which encompasses various physical, cognitive, behavioral, and emotional symptoms following a TBI. These symptoms can include headaches, dizziness, fatigue, insomnia, anxiety, irritability, and loss of memory or concentration [4,5]. A significant number of veterans with blast-related TBI report three or more PCS symptoms for up to three months after their injury occurs, and between 7.5% to 40% are affected by these chronic issues [2]. Additionally, those who suffer from repeated injuries due to trauma could progress to neurodegenerative diseases such as dementia which might become evident many years later [6].

The nature and setting of TBI in military personnel differ from that experienced by the civilian population. Military deployment to combat zones exposes service members to concussive blast hazards, such as improvised explosive devices (IEDs), suicide bombers, land mines, mortar rounds, and grenades. While TBIs sustained during deployments are significant, most recorded cases among military personnel occur outside these scenarios such as sports participation or vehicular accidents leading to falls [5]. Blast injuries are categorized into the following four mechanisms: primary, secondary, tertiary, and quaternary. An initial blast wound arises from the mechanical, thermal, and electromagnetic forces that emanate from an explosive mechanism. This generates a high-pressure wavefront that compresses the nearby air and quickly gives rise to negative pressure [5,7]. These compressed waves of pressure have the ability to travel at great speeds through various tissue types such as those with differing densities including air-fluid or fluid-solid structures which are particularly susceptible [8].

Apart from the primary physical injuries, consequential impairments in the form of secondary, tertiary, and quaternary damages can potentially affect brain functionality. Secondary injury may arise from shrapnel or other fragments propelled by a bomb blast [8]. The rapid acceleration of body parts via shock wave energy wind can result in tertiary damage that is comparable to coup-contrecoup injury [7]. Quaternary harm refers to all explosion-related wounds, diseases, or illnesses that cannot be attributed to previously mentioned mechanisms nor pre-existing medical conditions exacerbation. This encompasses burns, crush injuries, toxic inhalations, and cardiovascular complications, among others [5,8,9]. In addition to the four identified mechanisms of blast injury, there is a hypothesis that blast-related brain injuries can occur through various means. These include direct interaction with the head due to the passage of the blast wave through the skull and/or causing acceleration and rotation of the head, as well as transfer of kinetic energy from large blood vessels in abdominal and chest areas to central nervous system tissues. The transferred kinetic energy sets off an oscillatory wave that travels throughout body fluids at approximately a speed equivalent to sound in water, ultimately delivering this force directly to brain structures where it can cause both morphological and functional damage [7].

Numerous techniques have been employed in an endeavor to replicate TBIs caused by explosions. However, there are experimental obstacles associated with producing TBI-related occurrences in animal models. It is acknowledged that dependable animal models are imperative for comprehending the pathogenic mechanisms and devising novel therapeutic interventions for TBIs. Nonetheless, a clear set of criteria specifying trustworthy TBI animal models is lacking, which leads to inconsistencies among experimental approaches [7,10].

An academic investigation was conducted using a porcine model to explore the interplay between body and blast waves. The focus of the research was on low-level blast TBIs, specifically those incurred from exposure to gunner blasts [11]. Findings showed parenchymal microvascular alterations characterized by brain edema and vascular congestion as well as small hemorrhages in subarachnoidal and brain tissue areas within the occipital lobe, cerebellum, and medulla after subjecting the porcine model to gunner blasts [11]. Moreover, various trials have employed compressed-gas shock tubes or explosive-charged blast tubes to introduce living organisms into high-pressure environments. Animals such as rats and pigs are rendered unconscious and secured onto holders intended to restrict physical responses from the blasts and safeguard them against any tertiary effects of the shock or blast. Some studies also involve exposing animals in an open-field setting where detonated explosive blasts occur; however, this approach may yield a more varied array of biological reactions due to less manageable variables. Additionally, alternative experiments entail subjecting rabbits, rodents, sheep, or nonhuman primates to explosions for assessment purposes [7].

Due to ethical concerns surrounding animal experimentation, researchers have sought alternative methods for studying TBIs. One approach involves employing a skull-brain surrogate in conjunction with shock-tube blasts and fluid-structure interaction (FSI) simulations [12]. Computational simulations such as FSI enable healthcare professionals to visualize the physiological processes underlying both healthy and diseased states within the body. These simulations generate valuable information that can shed light on interrelationships among bodily systems while predicting treatment outcomes [13]. FSI finds applications in various scenarios such as modeling narrow vessels and heart valve closure. Additionally, it can also be used to study the interaction between cerebrospinal fluid (CSF) and the brain [14]. To conduct these analyses effectively using FSI methodologies in a scientific setting, researchers often rely on an arbitrary Lagrangian-Eulerian (ALE) formulation that requires dense meshes for accurate simulations [15]. However, difficulties arise using ALE when attempting to computationally simulate the contact between deformable solid structures and fluid domains with complex geometries commonly associated with human biology.

## Materials and methods

In 2012, IMPETUS Afea and the Norwegian Defense Research Establishment (FFI) established the anthropomorphic test device (ATD) project with the aim of creating a virtual ATD that could capture impulse and associated deformations during a blast event [16]. The utilization of ATDs in blast testing of combat vehicles is quite common as they serve as surrogates for the human body by representing military personnel’s average size. To calibrate the model, SAE International standards were followed while creating this complex model, which has been categorized into the following seven assemblies: head, arms, legs, feet upper torso lower torso, and neck to provide clarity on identifiable regions of ATD models [16].

ATD verification tests

The calibration of ATD models involved utilizing tests prescribed by SAE International [17] and the European Parliament [18], with the assistance of multiple companies such as IMPETUS Afea FFI and CertaSIM LLC. Nine calibration tests were performed to produce an accurate ATD suitable for crash test stimulation. These included the head drop test, neck flexion test, neck extension test, thorax impact test, knee impact test, knee slider test, upper foot impact test, lower foot impact test, and static foot impact test [16]. To ensure mass was factored into verifying blast event models, weight specifications from various assemblies were gathered from [17] and the output files [19]. Comprehensive elaborations on verification examinations and charts can be accessed from existing resources [16,19].

Mine blast testing

**Figure 1:**
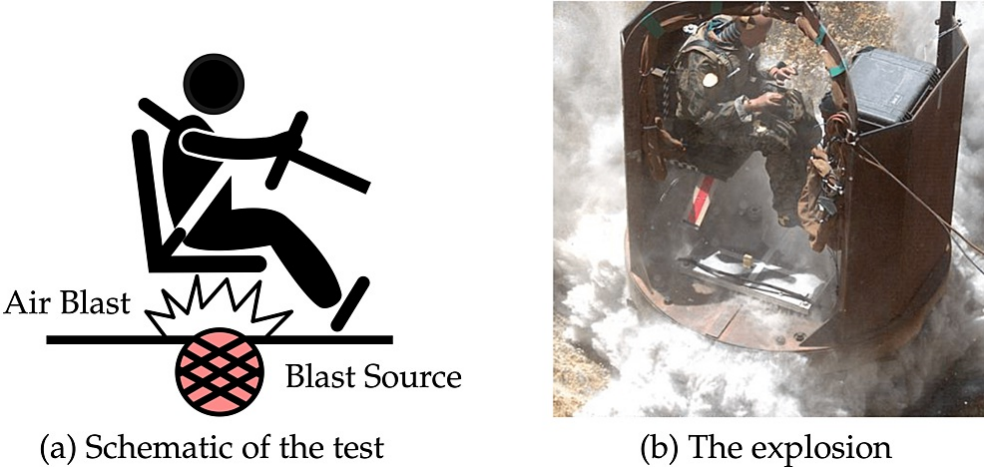
(a) Cartoon depiction of ATD position and blast source location during the experiment. (b) Image of ATD position and IED detonation during experimentation in South Carolina with the blast source under the reconstructed vessel. ATD = anthropomorphic test device; IED = improvised explosive device

At the General Dynamics Land Systems (GDLS) Edgefield Test Center in South Carolina, controlled and consistent blast survivability tests were conducted to verify numerical models [16] (Figure 1). To ensure accuracy, experiments were executed using specific procedures. The experimental matrix consisted of six test series that involved the ATD, a cage developed by GDLS, and two positions of the ATD’s tibia - angled at 90° and 110°. Mimicking documented blasts from an experimental database helped select charge type, size, shape, and depth for buried charges used during testing which further authenticated results obtained from previous tests. Repetitive trials were performed for each position due to lengthy setup times involving soil repacking with multiple firings possible within a day. Overall six well-documented tests were performed successfully as planned [19].

The configuration of the test includes a container that encloses various components such as the frame, floor, inflexible seat, ATD, and data acquisition apparatus (Figure 1). To achieve minimal flexure when transmitting loads to both the seat and floor while maintaining a rigid structure, thick-section steel is used for its composition [16]. The blast impact affects the ATD’s seated position via transmission through multiple layers including seat structure, cushioning material, and floor plate whereupon it rests their feet. A foam pad has been implemented on top of an unyielding chair with an additional thin layer behind the ATD’s back support for added protection during testing scenarios [19]. The use of a four-point harness maintains stability by securing the ATD to its seating arrangement; save for one exception in which an aluminum plate underneath remains somewhat flexible thus preventing total rigidity overall.

Multiple data channels were employed, which included conventional sensors embedded within the ATD and accelerometers on the testing apparatus. The test outcomes were recorded using two high-speed cameras mounted at different positions, i.e., one from above the tower of the testing rig and another from its side that captured results at 5,000 frames per second (fps) and 1,000 fps, respectively. Both filtered as well as unfiltered files of data were furnished for each experiment as it was necessary to utilize filtered values while displaying data [16]. Data points were obtained by utilizing tibia angles set up in two measurements, i.e., 90° and 110°, where four trials with a configuration angle of 90° followed by two experiments with an inclination angle measuring up to 110° were conducted during this study [19].

The testing conducted adhered to NATO Standard AEP-22 guidelines [20], which involved taking measurements of soil density and moisture content in the test pit. After each firing, the soil was excavated and repacked before proceeding with subsequent tests. The explosive utilized consisted of C4 charges arranged cylindrically with a diameter-to-height ratio of 3, while overburden soil was about 4 inches thick beneath the fixture [16]. Of concern were lumbar and tibia forces that occurred vertically as response parameters during experiments. Overall, findings revealed high reliability levels for experimental repeatability [16].

Mine blast verification

Jensen et al. [16] provided a comprehensive account of two simulations conducted using IMPETUS Afea Solver for modeling blast setup, which involved an ATD and fixture similar to those used in crash verification simulations. The numerical model accounted for gravity followed by transient dynamic blast events, with details on the simulation methodology explained in their research publications [16,19].

The primary objective of the simulation is to ensure that the ATD is appropriately positioned and its seat cushion adequately compressed before conducting tests. The IMPETUS Solver utilizes GPU technology for parallel processing, running on a workstation equipped with one NVIDIA Tesla K40c GPU. As a result, it took nearly nine hours to complete the gravity loading phase during simulations [16].

Moreover, the foundation of the blast simulation was established by recording and analyzing the fixture’s movement. The displacement of the seat during this occurrence was computed based on data gathered through a bottom accelerometer attached to it, which, in turn, served as an input for simulating ATD response without taking into account any structural responses from both the seat and floor frame [16]. During the initialization stage, first-run outcomes are incorporated within the seat model results implying that at time zero of the explosion event, ATD is seated while foam compression occurs concurrently with it being transiently dynamic over a 100 ms simulation period. By employing the aforementioned approach, approximately 7.5 hours were consumed to accomplish this simulation task effectively [19].

Boundary and initial conditions

**Figure 2:**
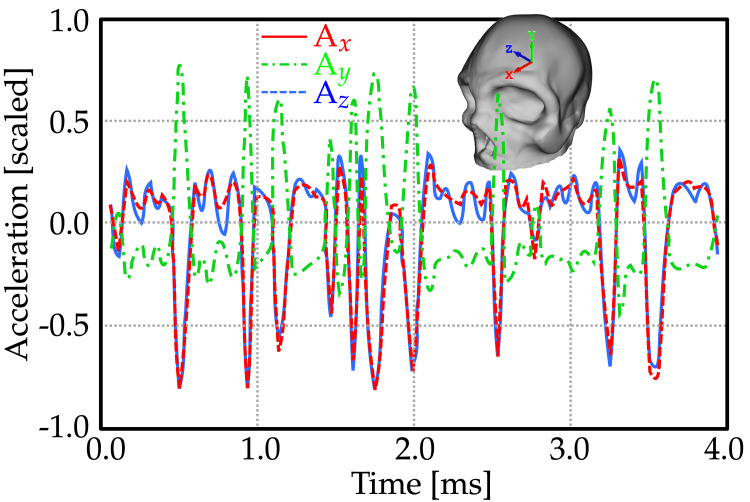
The experimental acceleration values prescribed to the skull.

The acquisition of four data sets pertaining to head and brain acceleration from CertaSIM, LLC in Saratoga, California was done. Each of the four sets was segregated according to X, Y, and Z directions for acceleration. One among these datasets was employed as a fixed standard for skull acceleration within the model used (Figure 2). After this step, calculations concerning fluid movement along with boundary interaction were resolved using IMPETUS Afea Solver employing smoothed-particle hydrodynamics (SPH) for fluid domain computations and high-order finite element method to solve solid-domain problems [21].

Geometrical model

**Figure 3:**
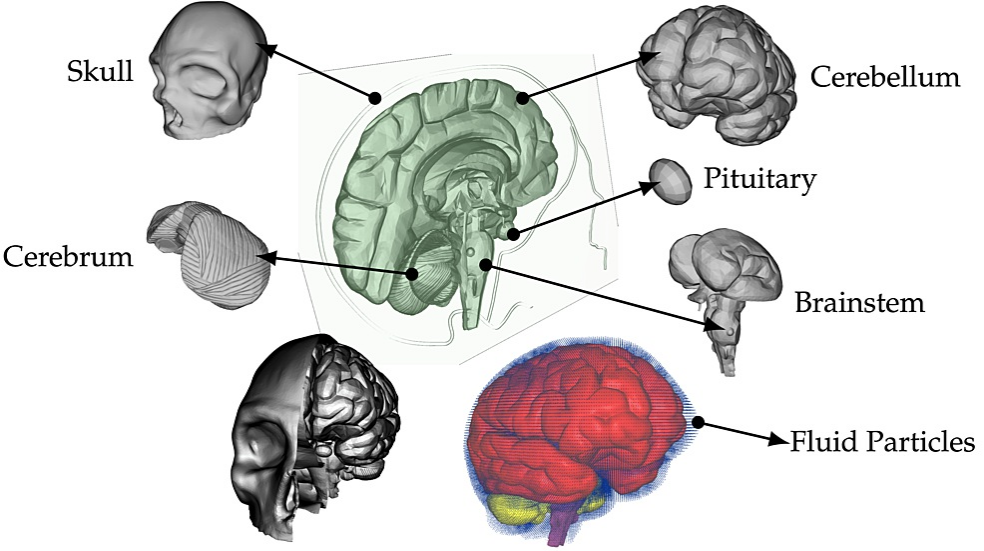
The illustration presents a comprehensive model of the head, featuring various components such as the skull, cerebrum, cerebellum, pituitary gland, and brain stem. Additionally, fluid particles occupying the subarachnoid space and other cavities are visualized through blue dots encompassing the depicted brain structure (lower right corner). Furthermore, half of the skull is also exhibited for clarity (left bottom side).

The model is composed of several distinct structures, such as the cerebrum, cerebellum, pituitary gland, brain stem, and skull. These components, each possessing individual material properties, are illustrated in Figure 3. In developing this patient-specific model, images were acquired from a DICOM-based online database. Consequently, some anatomical characteristics such as skin, meninges, and arachnoid granulations were omitted. Moreover, CSF flow was disregarded during simulations due to its relatively low flow rate of 0.05-0.08 ms^−1^ causing minimal displacement by only around 0.2-0.3 mm during short impact impulse times considered here; thus, negating any significance attributed to the presence of granules under these assumptions.

Smoothed-particle hydrodynamics

**Figure 4:**
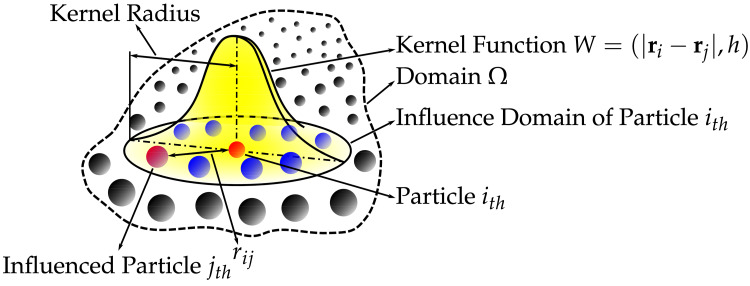
Smoothed-particle hydrodynamics kernel approximation.

The SPH technique, as demonstrated in Figure 4, was initially developed in 1977 as a mesh-free Lagrangian approach for astrophysical problem-solving [22]. In recent decades, it has become an effective tool for examining continuum media mechanics, including fluid flows and solid mechanics. Its versatility extends to various domains beyond astrophysics such as volcanology, ballistics, and oceanography, while gaining increasing acceptance among biomedical engineers [23,24]. A thorough explanation of the method’s formulation is available in our prior publications [25].

To simulate the interaction between fluid and solid domains, known as FSI, it is recommended to integrate high-order finite element methods with SPH methods, especially when intricate geometries are involved [26]. This combination is advantageous because SPH handles numerical contact between these domains easily and provides stability while being highly parallelizable. As a result of this hybrid approach, numerically stable and precise FSI simulations can be performed on standard GPU workstations instead of relying on expensive supercomputers. In addition to eliminating the need for geometry simplification, computation time is also significantly reduced from weeks or months to a relatively short duration.

## Results

**Figure 5:**
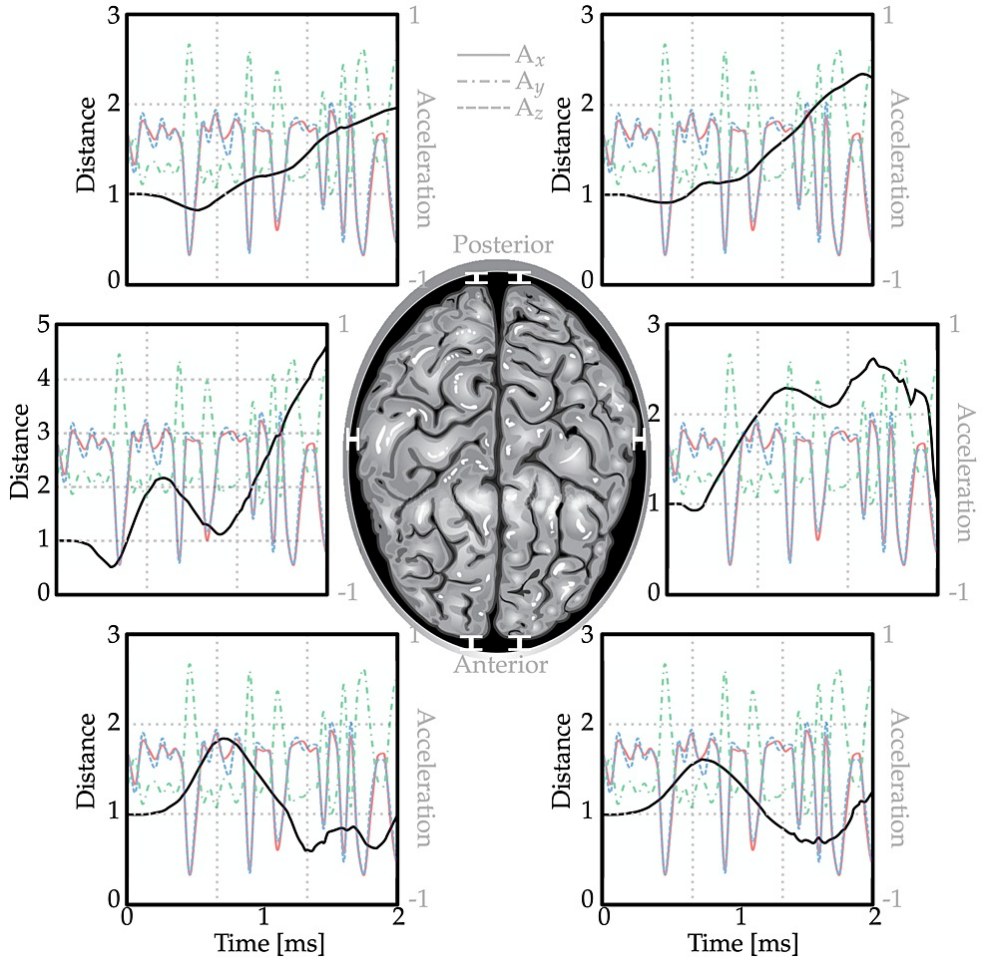
Normalized (starting from one) distances between the skull and brain in four different locations.

The results section presents a portrayal of the movement of the brain in relation to the skull under conditions of mine blast exposure, along with stress values applied on the brain. Additionally, it displays information regarding fluid particles and their corresponding movements. Figure 5 illustrates normalized distances between six distinct locations on both the skull and brain over time. Despite being subject to an oscillatory acceleration (also featured within these graphs), changes observed in distances between the brain and skull were comparatively less fluctuant, possibly attributable to CSF’s cushioning effects.

**Figure 6:**
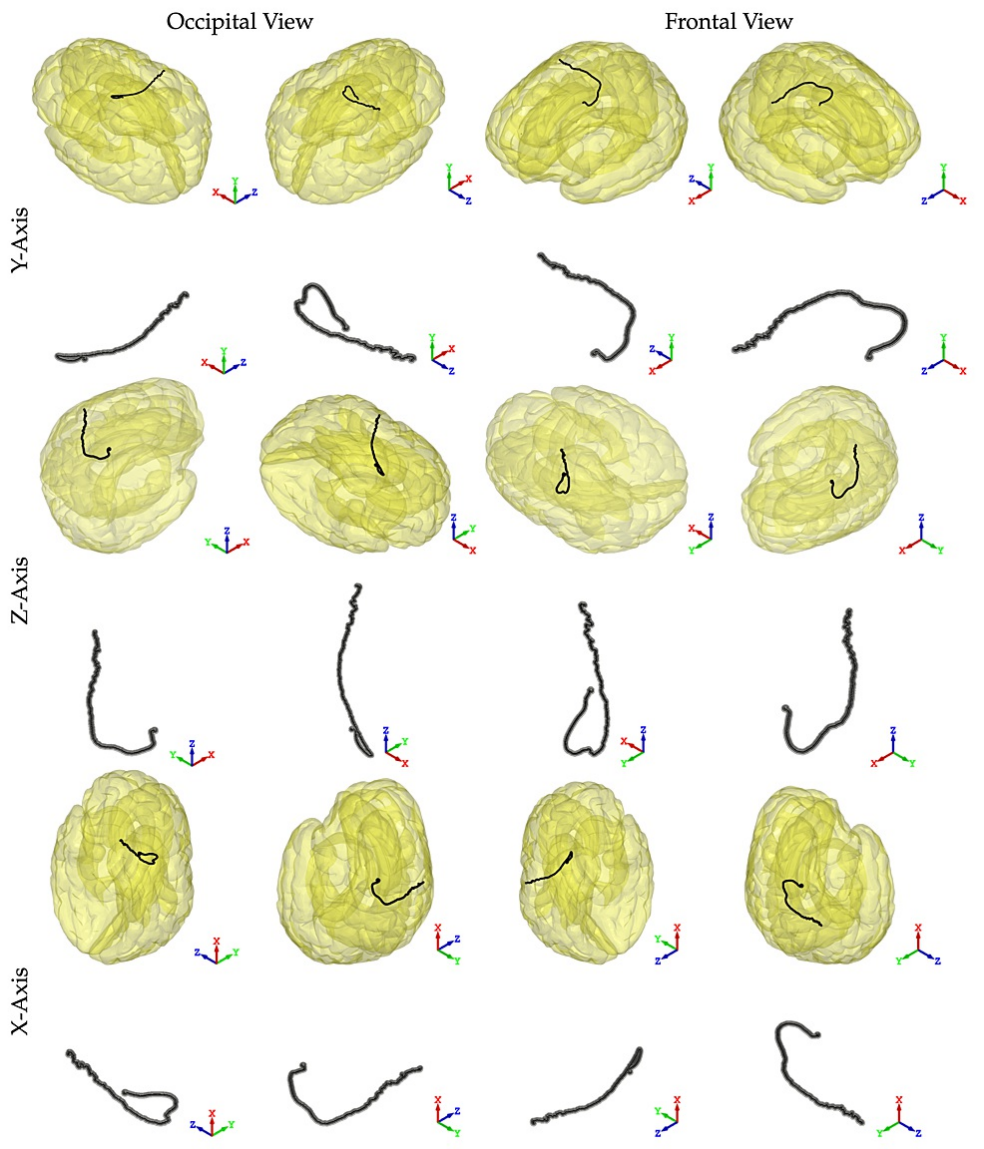
Simulation images of the right hemisphere simulation assessing the directionality of the brain during detonation in X, Y, and Z planes.

**Figure 7:**
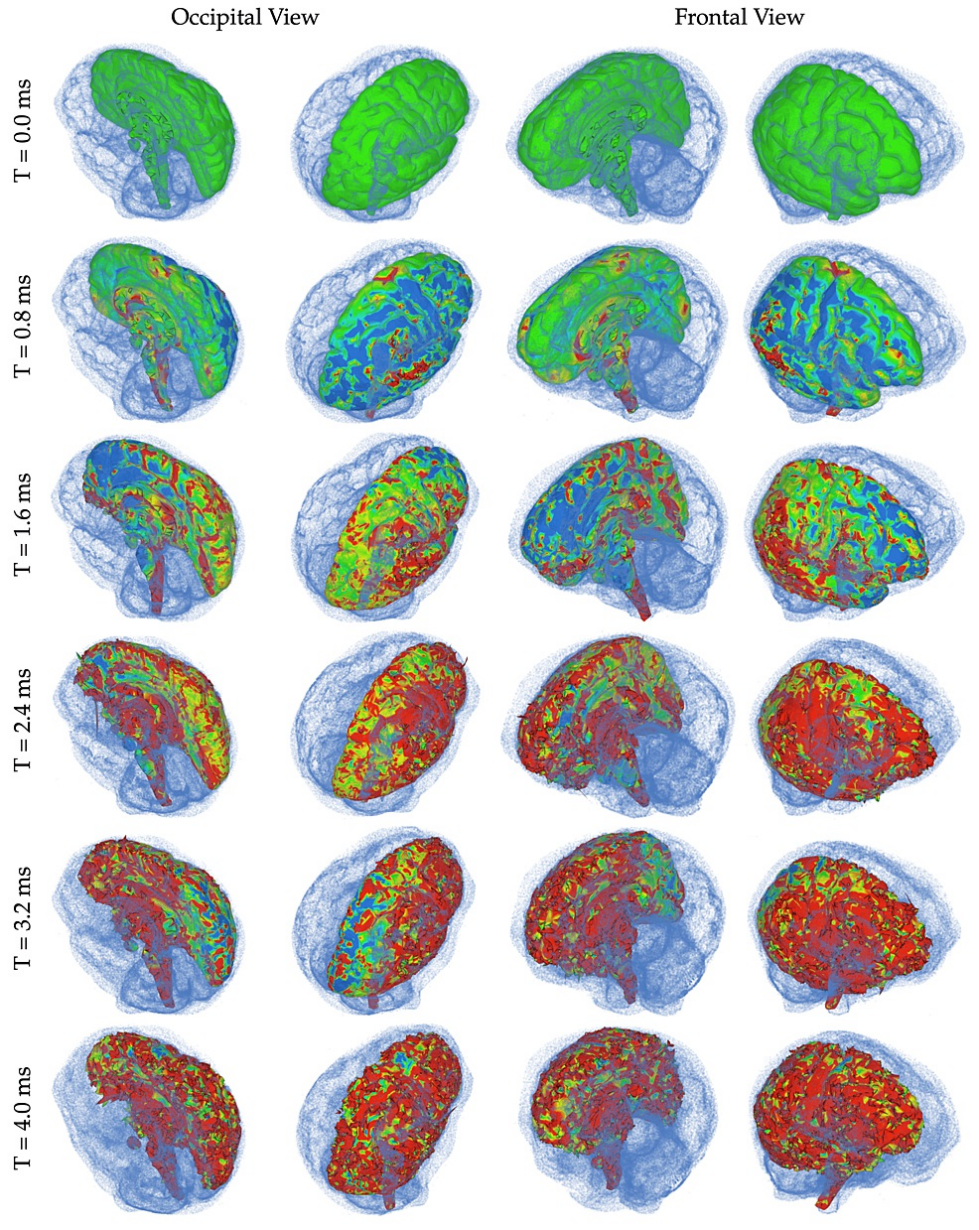
Simulation images of the right hemisphere simulation with surrounding fluid particles from the occipital and frontal views at different timepoints after detonation. Ranging from -3 MPa (blue) to +3 MPa (red).

To further illustrate the movement of the brain in relation to the skull during exposure to a mine blast, this study traces the path from a central point within the brain over time, as shown in Figure 6. The results indicate that significant movement primarily occurs in the horizontal plane (i.e., x-z plane depicted in Figure 6), with initial negative z-directional displacement followed by reversal toward positive z-directional motion. Notably, direct contact between the frontal lobe and skull was observed at T = 1.6 ms but not at T = 3.2 ms, as displayed in Figure 7. These findings can be explained by analyzing the directional movements of the brain presented in Figure 6.

Figure 7 illustrates the stress values (initial principal) of the brain, represented by blue and red color codes denoting -3 MPa and +3 MPa, respectively. The visualization offers insights from multiple viewpoints at different time instances. Additionally, further insights can be gleaned from the figure through the identification of regions where fluid particles are absent between the skull and brain relative to their initial state. For example, at T = 1.6 ms in the second column, a lack of fluid particles can be observed between the frontal lobe and skull when compared to its initial state at T = 0.0 ms (i.e., first row and second column).

Nevertheless, said fluid particles are still present in that location at both T = 0.8 ms and again at T = 3.2 ms; therefore, indicating that direct contact between the brain and skull occurred within this period of time. Taking into account positive stress values represent tension while negative ones represent compression, Figure 7 highlights a noteworthy event occurring at T = 1.6 ms whereby compressive stresses occur temporarily in the upper section of the frontal lobe as opposed to tensile stresses found throughout other areas such as right occipitoparietal region, which suggests contrecoup contusion might have occurred within the brain tissue.

## Discussion

The physiological implications of both tensile and compressive stresses experienced by the brain at specific time intervals are not yet fully understood. To gain a better understanding of the injury mechanisms associated with repeated directional changes to the head that occur closely together, further in-depth exploration is necessary. Experimental acceleration values assigned to the skull reveal multiple rapid changes in acceleration, resulting in a significant impact on the head. In a previous study, symptoms resembling shaken baby syndrome were observed under similar circumstances [27]. These findings, along with those from prior studies, indicate that while CSF surrounding the brain offers protection during initial shaking incidents, repeated episodes lead to direct contact between the skull and the brain.

Future advancements in the simulations could involve multiple iterations of major blood vessels and meninges located within the brain as well as the spinal cord, which have the potential to influence the underlying stiffness of the brain. Furthermore, simulation models can be developed by incorporating distinct volumes of CSF due to its average adult total volume ranging between 90 and 200 mL [28]. Varying CSF volumes among individuals require adjustments so that they are factored in accordingly for their respective alterations in simulating results concerning modeled scenarios regarding explosive-related injuries. In relation to the injury mechanism from an explosive device placed beneath a model, this particular simulation concentrates on such events; hence, forthcoming research should consider altering bomb placement orientation as injuries sustained may vary depending upon the blast wave’s directionality. With regards to blast waves’ orientations, future enhancements should enable replication effects resembling that of single blast-like waves using relevant iterations incorporated into this model. There is a need for additional investigation into the accuracy of the geometrical model to ensure that it accurately reflects real-life scenarios [23]. This research can pave the way for developing more sophisticated simulations that accurately depict the effects of explosive-related injuries on the brain and spinal cord. Overall, the continued refinement and validation of the geometrical model and associated simulations in predicting the effects of explosives on human anatomy are critical for advancing knowledge in the field of injury biomechanics and enhancing the effectiveness of protective measures for combat forces and civilians alike. Further research in this area would benefit from collaboration with medical professionals, including neurologists and anatomists, to ensure that the model closely aligns with the anatomical structure and function of the human brain and spinal cord, as well as its response to explosive-related injuries.

Due to the difficulty of studying blast-induced TBIs in vivo, simulations are necessary for investigating the prolonged implications of these injuries. The purpose of such simulations is to gain insight into how interactions between the brain, skull, and CSF result in severe disabilities among military personnel. While outcomes may vary from one individual to another, incorporating medical simulations into practices and diagnoses can enable better prediction of both short- and long-term effects resulting from blast-related trauma. By observing millisecond-by-millisecond changes within the brain through simulation technology, medical professionals will be better equipped to make informed decisions regarding interventions and therapies aimed at addressing TBI sequelae [29]. A geometrical model that simulates the interactions between the brain, skull, and CSF can provide valuable insights into blast-induced traumatic brain injuries and their consequences.

Enhanced comprehension of injury mechanisms may facilitate the formulation of protective measures, such as improving helmet design and other forms of safety equipment [30]. Additionally, simulations involving TBI can assist in evaluating cognitive impairments relevant to legal cases pertaining to individuals with TBI-related disabilities within the medicolegal system. This underscores the critical role that geometrical models and other simulation tools can play in furthering our understanding of TBI pathology, informing clinical decision-making, and driving innovation in the development of treatment options that are tailored to specific injury types and individual patient needs. Geometrical models that simulate blast-induced TBI can aid in the development of effective solutions aimed at preventing brain injuries while informing medical interventions and therapies for military personnel and civilians who have suffered from blast-related TBIs. Overall, simulations of blast-induced TBI hold great potential for advancing our understanding of these complex injuries and mitigating their effects. Hence, simulation studies can effectively be utilized as a scientific means to understand TBI mechanisms and their implications [14].

Moreover, the utilization of TBI simulations can facilitate virtual autopsies and afford a comprehensive perspective on the cerebral alterations stemming from diverse injury mechanisms such as cranial fractures or collisions. By providing a digital avenue for exploring TBI injury mechanisms, simulations can foster novel therapeutic techniques to impede the progression of TBIs. Consequently, we assert that TBIs instigated by blasts are arduous to scrutinize in vivo; thus, simulating their ramifications is essential. Enhancing the comprehension of these injuries’ mechanisms may promote strategies aimed at preventing TBIs.

In summary, the use of mathematical models to simulate blast-induced TBI holds immense potential for advancing scientific knowledge and developing innovative treatment options. It is imperative that further research be conducted to refine and validate these models, with the ultimate goal of translating findings into clinical practice. Future studies may examine the long-term outcomes of TBI-related disabilities in individuals with a history of blast exposure and the potential role that simulation tools can play in addressing these challenges and improving patient care. In closing, the promise of TBI simulations is significant and has tremendous potential to transform how we diagnose and treat these injuries.

## Conclusions

Experiments have demonstrated that there are frequent, rapid changes in acceleration values, which occur one after another and significantly impact the head. As a result of these sudden alterations, both tensile and compressive stresses are exerted on the brain within specific time intervals. However, our understanding of the physiological implications of these stresses is still limited. In a previous study, it was found that shaken baby syndrome exhibited similar characteristics to the circumstances being examined. The conclusion drawn from that investigation suggested that the CSF surrounding the brain only provides cushioning during an initial instance of shaking. However, in subsequent episodes of shaking, there is direct contact between the skull and brain observed. Our current research demonstrates a comparable occurrence where direct contact occurs after the initial peak of acceleration. This implies that CSF can only protect against damage during the first incident (the initial peak acceleration) but becomes ineffective thereafter. Based on our computational findings, multiple sudden changes in direction to the head cannot be prevented by CSF, potentially leading to brain damage.

To enhance our understanding of this phenomenon, we utilized a comprehensive geometric framework along with advanced computational algorithms to replicate these collisions and analyze their effects. This methodology allows for a more thorough comprehension of the mechanical strain distribution in various areas of the brain and can offer valuable insights into reducing the risk of injury, particularly for individuals who are exposed to repeated head impacts.
